# Association between plant-based diet quality and chronic kidney disease in Australian adults

**DOI:** 10.1017/S1368980024001095

**Published:** 2024-05-17

**Authors:** Jordan Stanford, Anita Stefoska-Needham, Kelly Lambert, Marijka J Batterham, Karen Charlton

**Affiliations:** 1 Nutrition and Dietetics Department, School of Medical, Indigenous and Health Sciences, University of Wollongong, Wollongong, NSW, Australia; 2 Illawarra Health and Medical Research Institute, Wollongong, NSW, Australia; 3 Director of the National Institute for Applied Statistics Research Australia and the Statistical Consulting Centre, School of Mathematics and Applied Statistics, University of Wollongong, Wollongong, NSW, Australia

**Keywords:** Plant-based diets, Dietary patterns, Dietary intake, 24-h recall, Chronic kidney disease, NNPAS 2011-13, Australia health survey

## Abstract

**Objective:**

To examine associations between three different plant-based diet quality indices, chronic kidney disease (CKD) prevalence and related risk factors in a nationally representative sample of the Australian population.

**Design:**

Cross-sectional analysis. Three plant-based diet scores were calculated using data from two 24-h recalls: an overall plant-based diet index (PDI), a healthy PDI (hPDI) and an unhealthy PDI (uPDI). Consumption of plant and animal ingredients from ‘core’ and ‘discretionary’ products was also differentiated. Associations between the three PDI scores and CKD prevalence, BMI, waist circumference (WC), blood pressure (BP) measures, blood cholesterol, apo B, fasting TAG, blood glucose levels (BGL) and HbA1c were examined.

**Setting:**

Australian Health Survey 2011–2013.

**Participants:**

*n* 2060 adults aged ≥ 18 years (males: *n* 928; females: *n* 1132).

**Results:**

A higher uPDI score was associated with a 3·7 % higher odds of moderate-severe CKD (OR: 1·037 (1·0057–1·0697); *P* = 0·021)). A higher uPDI score was also associated with increased TAG (*P* = 0·032) and BGL (*P* < 0·001), but lower total- and LDL-cholesterol (*P* = 0·035 and *P* = 0·009, respectively). In contrast, a higher overall PDI score was inversely associated with WC (*P* < 0·001) and systolic BP (*P* = 0·044), while higher scores for both the overall PDI and hPDI were inversely associated with BMI (*P* < 0·001 and *P* = 0·019, respectively).

**Conclusions:**

A higher uPDI score reflecting greater intakes of refined grains, salty plant-based foods and added sugars were associated with increased CKD prevalence, TAG and BGL. In the Australian population, attention to diet quality remains paramount, even in those with higher intakes of plant foods and who wish to reduce the risk of CKD.

Chronic kidney disease (CKD) is a growing burden to global health that affects 10–13 % of adults globally^(1)^. In Australia, one in ten (approximately 1·7 million) adults over the age of 18 years has indicators of CKD^(2–4)^. This burden of CKD is driven by conditions such as diabetes, high blood pressure (BP), high blood cholesterol and obesity^(2,5)^. Diet can help manage and reduce the risk of developing CKD and related conditions^(6)^. For example, plant-based diets have been associated with many relevant health benefits, including improved weight maintenance, insulin sensitivity and reduced blood pressure and cholesterol concentrations^(7–10)^. The protective effects of these diets are likely attributed to the synergistic effects of multiple nutrients provided by increased intakes of fruit and vegetables, legumes, whole grains, nuts and seeds, which are often lower in added sugars, salt, saturated fats, red meats and food additives^(11)^. Historically, international dietary guidelines for managing CKD have focused on modifying the amount of nutrients consumed, which ignores the complexity of diet and dietary behaviours. Without proper nutritional counselling, such nutrient restrictions may result in a low intake of nutritious plant foods^(12)^, limiting access to health-protective food components like dietary fibre and phytochemicals found naturally only in healthy plant foods.

Emerging observational evidence suggests a predominantly plant-based diet appears beneficial for people with CKD^(13–16)^, but risks differ by quality of plant-based diets. Limited research has specifically investigated this at the Australian population level. An analysis of population survey data comparing the degree to which people follow different plant-based diets and the association with CKD prevalence and health outcomes will provide further evidence of the potential significance of plant-based diets for CKD management in Australia. It will also allow insight into the current intake of the population, an essential consideration for translating findings into achievable recommendations.

Given the multitude of combinations of foods that can make up a plant-based diet, a series of different plant-based diet indices have been developed to differentiate between healthy and less healthy plant foods^(17,18)^. However, most of these current indices are only compatible with FFQ data, which often rely on estimations primarily derived from foods sourced entirely or predominantly from plants or animals. Such estimations overlook the inclusion of plant and animal ingredients in multi-ingredient foods and mixed dishes and disregard the context in which these ingredients are consumed. Recent research that has led to the development of a new food composition database^(19)^ identified large variability in the distribution of plant and animal ingredients across several food categories, including discretionary and core foods within the Australian food supply^(19)^. For example, although fruits, nuts and seeds are considered core foods themselves, they were more commonly present as ingredients in less healthy, discretionary products (such as cakes and confectionary) than in core foods^(19)^. Therefore, employing more accurate estimates and investigations that examine food sources at an ingredient level can pinpoint where the majority of plant sources are consumed, be used to provide more detailed information when elucidating the benefits of plant-based diets and may inform more targeted public health advice.

To address these gaps mentioned above, the present study aimed to evaluate the associations between three different plant-based diet quality indices, CKD prevalence and related risk factors, including anthropometric, biochemical and clinical measures, in a nationally representative Australian survey. The approach used to calculate plant-based diet quality index scores addresses limitations in the current evidence base by providing estimations of plant and animal content in the diet to the ingredient level. Moreover, it differentiates food ingredients consumed in distinct contexts, such as vegetables in a salad or pizza toppings.

## Methods

### Study design and populations

**Fig. 1:**
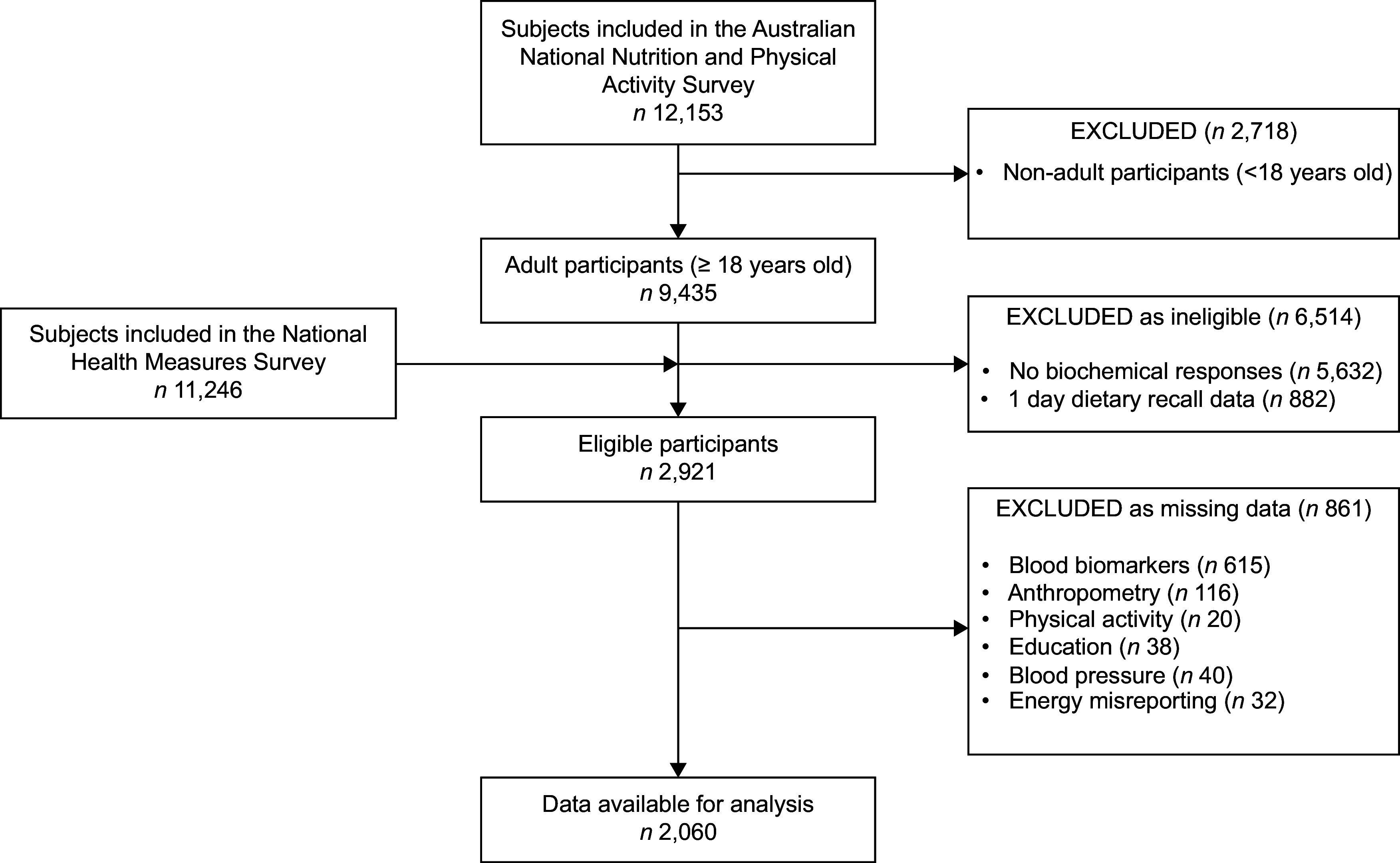
Eligible participants from the Australian Health Survey included in this secondary analysis

The 2011–13 Australian Health Survey (AHS) is Australia’s most recent and comprehensive nationally representative health survey, comprising three subcomponents. In addition to the 2011–2012 National Nutrition and Physical Activity Survey (NNPAS) and National Health Survey (NHS), the AHS includes a third component, the National Health Measures Survey, which invited participants aged five and older from both the NNPAS and NHS were invited to provide biological samples^(20)^ (Fig. 1). Over 9519 households across Australia took part in the NNPAS, collecting nutrition and dietary intake information from 12 153 children and adults aged between 2 and 85 years using 24-h dietary recalls^(20)^. Of these individuals, 7735 completed a second 24-h dietary recall (response rate: 63·6 %). Of the 30 329 respondents from the NHS and NNPAS, 11 246 (response rate: 37·1 %) participated in the National Health Measures Survey to provide blood and urine samples to measure nutritional status and chronic disease biomarkers^(20)^. Further details on the design and methodologies of these surveys are published elsewhere^(20)^.

The present study utilises data specifically from the NNPAS and the National Health Measures Survey. For this secondary analysis, data from individuals were excluded if they: (i) were less than 18 years old, (ii) only completed one 24-h dietary recall, (iii) were suspected of underreporting dietary intake based on implausible energy intakes (defined as an average daily energy intake <800 kcal/d for men and <500 kcal for women) (254, 255) and (iv) missing data for any covariate or variable of interest (Fig. 1). The STROBE-Nut statement (see online supplementary material, Supplemental Table S1) was used to facilitate comprehensive reporting of this study.

### Dietary intake assessment

As part of the survey, dietary intake data were collected through two separate 24-h recall diet assessments using an adapted version of the Automated Multiple Pass Method, an approach designed to maximise food recall and minimise memory bias^(21)^. The first 24-h recall was conducted face to face, while the second was collected at least 8 days after the first interview via telephone interview. The AHS survey user guide outlines additional details about the approach used within the questionnaire^(20)^.

Nutrient intakes were estimated from the reported intake data using the customised nutrient composition database developed by Food Standards Australia New Zealand, the AUSNUT 2011–2013 Food Nutrient Database^(22)^. For this study, the protein-to-fibre ratio was calculated by dividing the total dietary protein (g/d) by total dietary fibre (g/d)^(23,24)^. The potential renal acid load of the diet was determined by the established formula^(25)^: 0·49 × protein (g/d) + 0·037 × phosphate (mg/d) −0·021 × potassium (mg/d) − 0·026 × magnesium (mg/d) − 0·013 × calcium (mg/d).

### Plant-based dietary indices

Modified versions of three published a priori plant-based diet quality scores by Satija *et al.* ^(17)^ were employed. The three scores included (i) an overall PDI, (ii) a hPDI and (iii) an uPDI.

A recently developed Australian plant-based diet database^(19)^ compatible with the eight-digit codes of all the foods and beverages captured in the 2011–12 NNPAS was used to apply the diet quality indices. The database classifies total plant and animal intakes from single-, multi-ingredient foods and beverages and mixed dishes within the entire diet into twenty-three plant and animal food groups for each participant. ‘Healthy plant’ food groups include whole grains, fruits, vegetables, nuts and seeds, legumes, unsaturated plant oils/ spreads and tea and coffee, whereas ‘unhealthy plant’ foods include refined grains, fruit juices, saturated plant fats, sugars and syrups and miscellaneous plant products. ‘Animal’ food groups included animal fats, low-fat dairy, moderate-fat dairy, high-fat dairy, eggs, fish and seafood, processed or non-lean red meat and poultry, unprocessed, lean red meat and poultry and miscellaneous animal-based food items^(19)^. Further details relating to the database are provided in see online supplementary material, Supplemental Table S2 (Supplementary Materials). To take into account the context in which ingredients are consumed, for example, whole fruit as a snack *v*. fruit incorporated into a dessert – we employed a methodology similar to that described in detail elsewhere^(19)^. An additional scoring category was introduced to distinguish between the context in which plant or animal ingredients are consumed, whether they originate from ‘core’ (grains and cereals, vegetables, fruits, meat and alternatives, dairy and alternatives) or ‘discretionary’ (non-core) products or mixed dishes, aligning with the Australian Dietary Guideline definitions^(26)^. For this purpose, the Australian Health Survey: Users’ Guide, 2011–2013 – Discretionary Food List^(20)^ was used to classify foods and beverage items as core or discretionary.

Total intakes for each of the twenty-three food groups, according to core and discretionary products, were then calculated for both 24-h recalls, and the average of the 2 days was taken for each participant. In line with other studies using PDI scores^(17)^, we continued to use quintiles of consumption. Total average intakes for each of the twenty-three food groups from core and discretionary categories were then divided into quintiles of consumption, with each quintile being assigned a score. The overall PDI ranks diets according to the amount of plant-to-animal foods consumed, irrespective of the healthiness of the plant-based foods. When applying the overall PDI, participants received a score of five for each plant food group they consumed that equated to the highest quintile of consumption, a score of four for each plant food group consumed within the second-highest quintile, and so on, with a score of one for consumption below the lowest quintile (see online supplementary material, Supplemental Table S2). In contrast, the other two indices consider the healthiness of the plant foods consumed in addition to the quantity of intake. For instance, the hPDI scores diets higher that consist of more healthy plant foods, particularly if provided from the core foods group, whereas the uPDI allocates a higher score if diets contain more unhealthy plant foods, especially from discretionary food sources. Using the hPDI as an example, participants received a score of ten for each healthy plant food group consumed from core foods and a positive score of five for each healthy plant food group consumed from discretionary foods, if the participant was within the highest quintile of consumption. In contrast, participants were assigned reverse scores for each unhealthy plant food group consumed. Animal foods were assigned a reverse score for all three PDI scoring systems. Additional details regarding the scoring system for each index are provided in see online supplementary material, Supplemental Table S2. To obtain a final score for each diet quality index, the twenty-three food group scores were summed for each survey participant. Theoretical scores for the overall PDI were 46 (lowest possible score) and 230 (highest possible score), hPDI scores between 53 and 26, and uPDI scores between 51 and 255.

### Outcome measures

Trained and licensed phlebotomists collected urine and blood samples at pathology collection clinics or during a home visit^(20)^. These samples were used to assess nutritional status and chronic disease biomarkers, including kidney function and diabetes tests. CKD was defined based on the participants’ estimated glomerular filtration rate, together with their urinary albumin creatinine ratio^(20)^. Individuals considered not to have CKD were those with an estimated glomerular filtration rate > 60 ml/min and no albuminuria. CKD stages 1 and 2 are diagnosed by the presence of albuminuria, regardless if their estimated glomerular filtration rate was greater than 60 ml/min. The remaining three stages (CKD stages 3–5) are determined according to an estimated glomerular filtration rate of less than 60 ml/min^(20)^. For the present analysis, participants were classified as having CKD if they had stage 1–5 indicators, while moderate-severe CKD was defined as individuals with indicators of CKD stages 3–5. Additional biochemical outcomes of interest included total blood cholesterol concentrations, HDL-cholesterol and LDL-cholesterol (mmol/l), fasting TAG (mmol/l), apo B (Apo B) (g/l), fasting blood glucose (mmol/l) and Hb A1c (HbA1c) (%). Results for LDL-cholesterol, TAG and plasma glucose were obtained from participants who had fasted for at least 8 h prior to providing a blood sample. Apo B, HbA1c, total cholesterol and HDL cholesterol were measured in biological samples without the need for fasting.

As part of the initial AHS interviews, anthropometric measurements, such as waist circumference (cm), weight (kg) and height (cm), were taken by trained personnel using tape measures, digital scales and stadiometers, respectively^(20)^. BMI was calculated using Quetelet’s metric (kg/m^2^). The main clinical parameter of interest in this study was blood pressure. Systolic blood pressure and diastolic blood pressure measurements were taken on the left arm using an automated blood pressure monitor^(20)^. A third measurement was taken in cases where the systolic and diastolic pressures differed more than 10 mmHg. When only two readings were needed, the second reading was used for systolic and diastolic pressure measures. If a third reading was needed, the average of the second and third readings was used. Additional details regarding the methodology used to collect anthropometric, biochemical and clinical measures are described elsewhere^(20)^.

### Covariates

CKD risk factors^(5)^ or variables known to influence outcome measures of interest were important covariates included in our regression models. Socio-demographic and lifestyle characteristics were collected via interviewer-administered questionnaires (244). Socio-demographic characteristics included age, sex and education status, which were classified as high (having a University qualification), medium (completed high school or completed some high school and/or certificate/diploma) and low (completed some high school or less)^(20)^. As per the ABS classification, lifestyle characteristics included smoking status was categorised as never smoked, ex-smoker or current smoker and physical activity level, which was categorised as high, moderate, low and sedentary, determined by the intensity and duration of the activity undertaken by the participant^(20)^. Comorbidities defined based on certain clinical and biochemical measurements (outlined in section 5·2·4) were also included. For example, hypertension was classified as having blood pressure greater than or equal to 140/90 mmHg^(20,27)^. Diabetes risk was defined based on HbA1c, where an HbA1c <6 % was considered normal, 6–6·4 % considered at risk of diabetes and ≥ 6·5 % indicates diabetes^(20)^. Anthropometric measures such as BMI and dietary factors obtained from 24-h recalls, including total energy and alcohol intake, were also included as covariates.

### Statistical analyses

To generalise the results to the Australian population at the time of the survey, we used the complex survey design method, which incorporates sampling and replicate weights. Survey weightings were calibrated against population benchmarks designed by the ABS to account for bias associated with those volunteers who provided biological samples^(20)^. All analyses were conducted using Stata (StataCorp Stata Statistical Software, College Station TX: Release 15, 2017).

Demographic characteristics were summarised according to quintiles of each PDI index (overall PDI, hPDI and uPDI). The number of unweighted participants (n) in each quintile varies since quintiles represent weighted distributions of participants. Continuous variables were analysed using linear regression modelling, and adjusted means were calculated for each PDI index. P values were calculated for linear trends across groups. Categorical variables were analysed through Pearson’s χ2 with an overall significance determined as *P* < 0·05, while a significant difference between the groups was determined at *P* < 0·005 through individual Pearson’s χ2 analysis with a Bonferroni correction for multiple comparisons.

Logistic regression models were conducted to determine the associations between PDI scores and biomarkers of CKD. Linear regression analyses were also conducted to determine the associations for each PDI index with biochemical, anthropometric and clinical measures. Normality and descriptive statistics of each outcome variable were assessed, as were the normality of residuals, homoscedasticity and linearity for each regression analysis. The natural log values for HDL-cholesterol, fasting TAG, fasting blood glucose concentrations, HbA1c, systolic blood pressure, WC and BMI were used due to non-normal distributions. Regression models and confounding variables differed depending on the outcome variable of interest. For example, the simple regression model adjusted for age, sex and energy intakes (kJ/d). Multivariate models further adjusted for various socio-demographic characteristics (i.e. education status), dietary factors (i.e. alcohol intake), BMI, lifestyle factors (i.e. physical activity and smoking status) and comorbidities (i.e. hypertension and diabetes). Except for BMI and waist circumference, all other outcome analyses were adjusted for BMI. Table footnotes provide specific information of the covariates used in each regression model. Statistical significance was indicated as *P* < 0·05.

## Results

A total of 2060 individuals were included in the present analysis (men: *n* 928; women: *n* 1132) (Fig. 1). Twelve per cent (*n* 250) of these participants had CKD, of which 41·2 % (*n* 103) had moderate to severe CKD.

Respondent characteristics according to quintiles of each PDI index (overall PDI, hPDI and uPDI) are shown in Table 1. Participants within the highest quintile of the overall PDI were less likely to smoke than those in other quintiles (*P* < 0·05). The hPDI was positively associated with age (*P* < 0·01), while university qualification differed between quintiles (*P =* 0·02). Compared with those in the lowest quintile, a higher proportion of individuals had indicators of diabetes within the highest quintile (*P* < 0·05). Finally, those with a greater uPDI score were more likely to be younger (*P* < 0·01) and smoke (*P* < 0·01).

**Table 1:** Characteristics of study participants according to overall PDI, healthy PDI and unhealthy PDI scores* ^,^ †

					Overall PDI							
	Q1 *n* 2 043 842 (unweighted *n* 459)		Q2 *n* 1 589 789 (unweighted *n* 379)		Q3 *n* 1 921 915 (unweighted *n* 445)		Q4 *n* 1 794 994 (unweighted *n* 449)		Q5 *n* 1 419 445 (unweighted *n* 328)			
	x̅ or %	se	x̅ or %	se	x̅ or %	se	x̅ or %	se	x̅ or %	se	*P* value for linear trend‡	*P* value for significant difference‡
Age (years)§,¶	43·34*	1·29	46·42*	1·33	48·24*	1·79	46·80*	1·28	47·47*	1·70	0·14	0·20
Female (%)||	55·44*	3·33	53·96*	3·69	51·33*	4·03	48·71*	3·71	47·85*	4·05	–	0·59
University qualification (%), High||	33·55*	3·47	26·20*	3·71	29·37*	3·99	32·17*	3·99	35·07*	4·66	–	0·62
Current smoker (%)||	12·19*	2·04	12·79*	2·61	14·06*	2·92	12·21*	2·98	2·52	1·08	–	0·09
Physical activity, High (%)||	17·41*	2·67	13·68*	2·90	17·08*	3·74	14·18*	2·96	26·48*	3·84	–	0·41
Met physical activity guidelines (%)||,**	55·76*	3·88	52·75*	4·01	57·23*	3·85	54·60*	4·36	61·43*	3·95	–	0·67
Diabetes (%)||	3·19*	0·79	4·62*	1·13	4·97*	1·08	4·65*	1·16	7·34*	1·91	–	0·50
Hypertension (%)||	19·65*	2·95	19·51*	3·04	17·18*	2·74	19·72*	2·80	15·74*	2·82	–	0·82
Alcohol (g/d)§,* ‡	14·62*	1·49	15·53*	1·80	12·28*	1·41	12·00*	1·26	10·28*	2·02	0·06	0·29
Energy (kJ/d per kg)§,*†	115·99*	4·01	116·98*	4·08	121·00*	4·73	123·65*	3·70	144·95	6·12	**<0·01**	**<0·01**
Protein (g/d per kg)§,*‡	1·28*	0·04	1·30*	0·04	1·23*	0·04	1·24*	0·03	1·24*	0·04	0·24	0·60
Total fat (g/d)§,*‡	80·26†	1·42	74·23*	1·33	76·15*,†	1·82	74·15*	1·65	77·30*,†	2·67	0·35	**0·02**
Saturated fat (g/d)§,*‡	32·47	0·64	28·66*	0·61	28·05*	0·80	26·78*	0·72	25·75*	0·94	**<0·01**	**<0·01**
Monounsaturated fats (g/d)§,*‡	29·64*	0·65	28·21*	0·59	29·47*	0·90	28·38*	0·75	30·50*	1·31	0·53	0·24
Polyunsaturated fats (g/d)§,*‡	11·37*	0·36	10·89*	0·42	12·16*,†	0·35	12·43*,†	0·43	14·32†	0·76	**<0·01**	**<0·01**
Dietary fibre (g/d)§,*‡	19·96	0·43	22·51*	0·66	24·73*,†	0·69	26·52†	0·52	31·39	0·78	**<0·01**	**<0·01**
Na (mg/d)§,*‡	2558·20*	57·46	2345·79*	62·98	2529·96*	102·84	2330·15*	70·83	2331·34*	57·60	**0·02**	**<0·05**
Potassium (mmol/d per kg)§,*‡	0·95*	0·03	1·04*,†,‡	0·04	1·04*,†	0·03	1·09†,‡	0·02	1·19‡	0·04	**<0·01**	**<0·01**
Mg (mg/d)§,*‡	341·58*	8·68	355·18*,†	9·38	366·29*,†	10·08	376·41†	7·96	434·23	15·74	**<0·01**	**<0·01**
Ca (mg/d)§,*‡	905·04*	29·74	943·00*	47·78	902·33*	40·87	891·58*	28·78	903·65*	33·06	0·58	0·92
Phosphorous (mg/d)§,*‡	1580·73*	33·42	1541·49*	45·16	1517·30*	41·33	1477·92*	20·61	1543·79*	43·08	0·26	0·12
Protein: fibre ratio§,*‡,*§	5·36	0·15	4·69†	0·14	4·16*,†	0·16	3·66*	0·10	3·09	0·10	**<0·01**	**<0·01**
PRAL§,*‡	25·98	1·36	19·13†	1·35	16·20*,†	1·93	12·45*	1·30	7·95*	1·95	**<0·01**	**<0·01**
					Healthy PDI							
	Q1 *n* 1 840 744 (unweighted *n* 372)		Q2 *n* 1 766 008 (unweighted *n* 406)		Q3 *n* 1 897 609 (unweighted *n* 436)		Q4 *n* 1 640 102 (unweighted *n* 428)		Q5 *n* 1 625 523 (unweighted *n* 418)			
	x̅ or %	se	x̅ or %	se	x̅ or %	se	x̅ or %	se	x̅ or %	se	*P* value for linear trend‡	*P* value for significant difference‡
Age (years)§,¶	38·45	1·33	46·71*	1·57	46·21*	1·66	48·05*	1·48	53·34*	1·52	**<0·01**	**<0·01**
Female (%)||	47·56*	3·75	56·04*	3·61	46·80*	4·02	50·50*	4·46	58·42*	3·72	–	0·20
University qualification (%), High||	38·06*	3·39	22·01†	2·85	26·39*,†	3·72	32·12*,†	3·90	38·43*	4·31	–	**0·02**
Current smoker (%)||	11·06*	2·90	13·53*	2·52	10·25*	2·28	11·25*	3·41	9·60*	2·38	–	0·96
Physical activity, high (%)||	16·33*	3·43	13·08*	2·82	18·01*	4·13	19·49*	3·69	20·84*	3·46	–	0·82
Met physical activity guidelines (%)||,**	53·87*	4·23	52·79*	4·38	54·85*	5·02	58·21*	3·11	62·18*	3·70	–	0·51
Diabetes (%)||	1·64*	0·56	4·63*,†	1·59	4·89*,†	1·12	4·68*,†	1·30	8·63†	1·89	–	0·07
Hypertension (%)||	13·88*	2·69	19·98*	2·84	18·67*	3·08	17·54*	2·50	22·72*	3·25*	–	0·29
Alcohol (g/d)§,* ‡	11·07*	1·25	15·62*	1·83	13·23*	1·69	13·44*	1·68	11·82*	1·50	0·90	0·31
Energy (kJ/d per kg)§,* †	129·98†	3·92	130·04*,†	4·22	120·14*,†	4·00	114·09*	3·81	122·58*,†	6·27	0·06	**0·03**
Protein (g/d per kg)§,* ‡	1·22*	0·05	1·27*	0·05	1·26*	0·03	1·28*	0·04	1·27*	0·03	0·48	0·93
Total fat (g/d)§,* ‡	76·68*	1·80	77·57*	1·64	77·40*	1·93	73·85*	1·51	76·96*	2·51	0·62	0·28
Saturated fat (g/d)§,* ‡	31·84‡	0·87	30·28‡	0·71	29·09†,‡	1·01	26·09*,†	0·65	24·84*	0·70	**<0·01**	**<0·01**
Monounsaturated fats (g/d)§,* ‡	27·77*	0·74	29·00*	0·75	29·69*	0·83	28·93*	0·75	30·86*	1·36	0·07	0·24
Polyunsaturated fats (g/d)§,* ‡	10·51*	0·42	11·64*,†	0·44	11·93*,†	0·39	12·36†,‡	0·33	14·60‡	0·76	**<0·01**	**<0·01**
Dietary fibre (g/d)§,* ‡	18·66	0·46	22·07	0·45	25·29*	0·77	26·34*	0·67	31·84	0·70	**<0·01**	**<0·01**
Na (mg/d)§,* ‡	2710·96‡	91·73	2474·80†,‡	58·49	2503·31†,‡	107·10	2324·71*,†	69·43	2084·43*	50·99	**<0·01**	**<0·01**
Potassium (mmol/d per kg)§,* ‡	0·89*	0·03	1·03*,†	0·04	1·07†	0·03	1·09†	0·03	1·21	0·04	**<0·01**	**<0·01**
Mg (mg/d)§,* ‡	304·63	9·03	344·24*	7·07	377·26*,†	9·29	391·66†	10·23	450·24	11·88	**<0·01**	**<0·01**
Ca (mg/d)§,* ‡	870·26*	40·38	893·07*	37·81	958·90*	43·80	880·75*	32·92	936·90*	28·18	0·26	0·47
Phosphorous (mg/d)§,* ‡	1487·51*	44·72	1492·48*	38·79	1573·44*	39·94	1535·56*	34·51	1577·10*	38·09	0·09	0·41
Protein: fibre ratio§,* ‡,* §	5·34	0·18	4·55†	0·10	4·21*,†	0·15	3·93*	0·18	3·10	0·11	**<0·01**	**<0·01**
PRAL§,* ‡	25·56	1·56	18·45*	1·22	16·59*	1·78	15·31*	1·58	7·41	2·11	**<0·01**	**<0·01**
	Unhealthy PDI											
	Q1 *n* 1 840 744 (unweighted *n* 455)		Q2 *n* 1 766 008 (unweighted *n* 493)		Q3 *n* 1 897 609 (unweighted *n* 404)		Q4 *n* 1 640 102 (unweighted *n* 332)		Q5 *n* 1 625 523 (unweighted *n* 376)			
	x̅ or %	se	x̅ or %	se	x̅ or %	se	x̅ or %	se	x̅ or %	se	*P* value for linear trend‡	*P* value for significant difference‡
Age (years)§,¶	47·93*	1·57	47·89*	1·30	48·00*	1·47	45·37*	1·92	42·07*	1·52	**0·01**	0·06
Female (%)||	45·19*	3·91	50·14*	3·32	55·76*	3·90	55·20*	3·88	52·90*	4·45	–	0·36
University qualification (%), high||	36·01*	3·31	33·90*,†	3·46	33·30*,†	4·17	24·17†	4·39	27·54*,†	4·00	–	0·09
Current smoker (%)||	6·41*	1·60	9·24*	1·79	7·72*	1·93	14·46*,†	2·51	18·80†	3·75	–	**<0·01**
Physical activity, high (%)||	21·50*	3·58	14·63*	2·74	17·98*	4·54	20·73*	3·67	13·21*	2·82	–	0·40
Met physical activity guidelines (%)||,**	64·32*	3·35	53·18*	4·23	55·34*	4·42	56·95*	4·85	51·66*	4·29	–	0·26
Diabetes (%)||	3·02*	0·87	4·45*	0·86	6·39*	1·61	6·12*	1·65	4·32*	1·27	–	0·82
Hypertension (%)||	23·64*	4·15	15·56*	2·06	18·38*	2·37	17·87*	2·87	17·19*	2·17	–	0·28
Alcohol (g/d)§,* ‡	15·08*	2·09	10·69*	1·15	13·14*	1·45	12·45*	1·61	14·13*	1·43	0·98	0·38
Energy (kJ/d per kg)§,* †	135·84†	4·22	125·55†	2·70	122·37*,†	5·93	125·41*,†	5·40	107·78*	4·05	**<0·01**	**<0·01**
Protein (g/d per kg)§,* ‡	1·35†	0·03	1·31†	0·04	1·22*,†	0·04	1·26*,†	0·05	1·13*	0·04	**<0·01**	**<0·01**
Total fat (g/d)§,* ‡	84·60†	1·30	80·69†	1·97	78·55†	2·18	70·79*	1·33	66·36*	1·36	**<0·01**	**<0·01**
Saturated fat (g/d)§,* ‡	31·18‡	0·91	30·04‡	0·82	28·78†,‡	0·80	26·84*,†	0·60	25·38*	0·76	**<0·01**	**<0·01**
Monounsaturated fats (g/d)§,* ‡	32·35†	0·51	30·81†	0·90	30·64†	1·13	26·60*	0·60	25·02*	0·62	**<0·01**	**<0·01**
Polyunsaturated fats (g/d)§,* ‡	13·69‡	0·47	12·79†,‡	0·39	12·52*,†,‡	0·69	11·26*,†	0·43	10·20*	0·40	**<0·01**	**<0·01**
Dietary fibre (g/d)§,* ‡	27·04‡	0·59	26·14‡	0·77	25·49†,‡	0·84	22·74*,†	0·72	21·32*	0·57	**<0·01**	**<0·01**
Na (mg/d)§,* ‡	2574·21†	72·82	2581·08*,†	90·06	2364·89*,†	63·43	2349·13*,†	65·59	2239·22*	71·73	**<0·01**	**<0·01**
Potassium (mmol/d per kg)§,* ‡	1·14*	0·03	1·10*	0·03	1·07*	0·05	1·03*	0·03	0·91	0·02	**<0·01**	**<0·01**
Mg (mg/d)§,* ‡	420·18†	8·22	393·09†	8·13	384·20*,†	13·64	342·75*	9·36	308·78	6·28	**<0·01**	**<0·01**
Ca (mg/d)§,* ‡	999·80†	32·07	985·35*,†	34·79	934·28*,†	41·18	863·96*	28·99	735·47	28·46	**<0·01**	**<0·01**
Phosphorous (mg/d)§,* ‡	1705·58‡	32·26	1633·14†,‡	28·17	1523·93*,†	34·46	1466·09*	36·05	1302·36	28·30	**<0·01**	**<0·01**
Protein: fibre ratio§,* ‡,* §	4·07*	0·12	4·28*	0·16	4·06*	0·15	4·43*	0·20	4·49*	0·20	**0·04**	0·23
PRAL§,* ‡	19·19*	1·53	17·38*	1·82	13·74*	1·63	17·66*	1·71	16·49*	1·49	0·31	0·12

PDI, plant-based diet index; PRAL, potential renal acid load.

* *N* 8 769 986 (unweighted *n* 2060). Sampling and replicate weights were used to generalise the results to the Australian population at the time of the survey.

† Categories sharing capital letters within rows are not statistically significant from each other. Comparison of means was conducted through pairwise comparison. All comparisons applied a Bonferroni correction for multiple comparisons such that a significant difference was observed at *P* < 0·005.

‡ Bolded *P* values denote those that are statistically significant *P* < 0·05

§ Reported as x̅ (sem)

|| Reported as percentage (se).

¶ Survey linear regression adjusted for sex.

** Australian physical activity recommendation of 150 min of moderate exercise per week.

*† Survey linear regression adjusted for age and sex.

*‡ Survey linear regression adjusted for age, sex and energy intake (kJ/d).

*§ *Protein: fibre ratio*, total dietary protein (g/d) divided by total dietary fibre (g/d).

Energy intakes were reportedly higher for those with a greater overall PDI score (*P* < 0·01) and lower for the uPDI (*P* < 0·01). Individuals with higher scores for both the PDI and hPDI reported higher intakes of polyunsaturated fats, dietary fibre, Mg and potassium (all *P* < 0·01), while lower intakes of saturated fat (*P* < 0·01), Na (*P* < 0·05), protein to fibre ratio and potential renal acid load score (both *P* < 0·01). Participants with greater scores for the uPDI reported lower intakes of all nutrients presented in Table 1 (*P* < 0·01). The uPDI also revealed a linear trend towards a higher protein-to-fibre ratio in the diet.

**Fig. 2:**
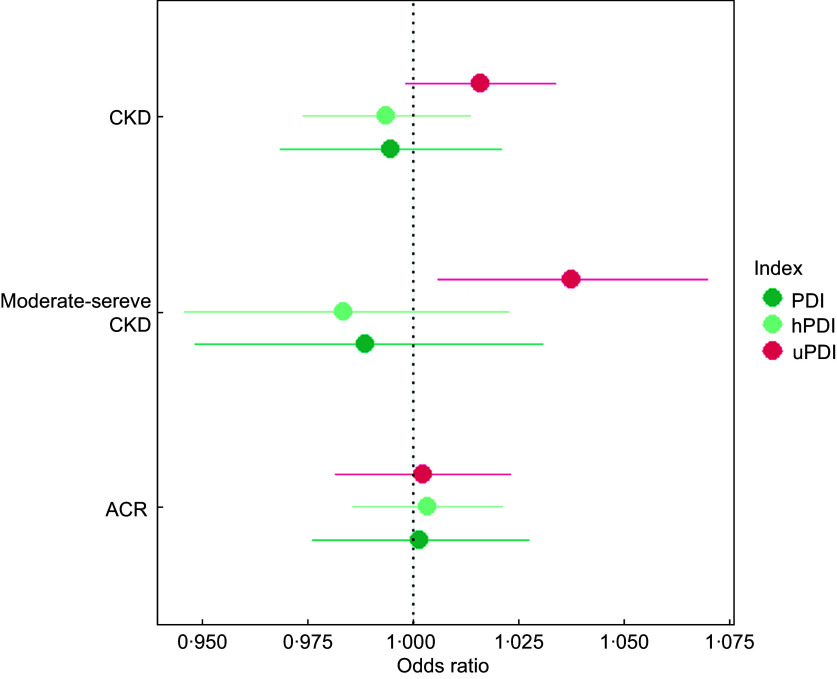
Association between plant-based diet quality and CKD prevalence and severity in Australian adults adjusted for age, sex, intake of energy, education, physical activity, smoking status, diabetes, hypertension, BMI and intake of alcohol. N 8 769 986 (unweighted n 2060). Sampling and replicate weights used to generalise the results to the Australian population at the time of the survey. ACR, albumin-to-creatinine ratio; CKD, chronic kidney disease; PDI, overall plant-based diet index; uPDI, unhealthy plant-based diet index; hPDI, healthy plant-based diet index

For all respondents, a higher uPDI score was associated with 1·7 % increased odds of CKD and 3·5 % higher odds of moderate-severe CKD. However, after correcting for socio-demographic characteristics, BMI, dietary, lifestyle and comorbidities, only the OR for moderate-severe CKD remained statistically significant and increased to 3·7 % (95 % CI: 1·006–1·07) (Fig. 2 and see online supplementary material, Supplemental Table S3). No significant associations between higher PDI or hPDI scores and CKD prevalence were observed.

Associations between the three PDI scores, anthropometric, biochemical and clinical measures are summarised in Table 2. A greater overall PDI score was inversely associated with waist circumference (*P* < 0·001), whereas both the overall PDI and hPDI were inversely associated with BMI (*P* < 0·001 and *P* = 0·0185, respectively). The overall PDI score was also related to lower systolic and diastolic BP. Although for diastolic BP, the statistical significance was lost after adjusting for relevant socio-demographic characteristics and lifestyle factors (*P* = 0·091). Interestingly, a higher uPDI score was inversely associated with total blood cholesterol, LDL-cholesterol and HDL-cholesterol concentrations. However, the association for HDL-cholesterol did not remain statistically significant after accounting for socio-demographic and lifestyle factors. Finally, a higher uPDI was associated with increased fasting TAG (*P* = 0·0323) and blood glucose concentrations (*P* < 0·001), even after adjusting for relevant socio-demographic characteristics, dietary and lifestyle factors.

**Table 2:** Associations between plant-based diet quality indices and anthropometric, biochemical and clinical risk factors in Australian adults*

		Overall PDI score				Healthy PDI score				Unhealthy PDI score			
	Model	Coef.	95 % Conf. Interval		*P* value†	Coef.	95 % Conf. Interval		*P* value†	Coef.	95 % Conf. Interval		*P* value†
Total cholesterol (mmol/l)	1	−0·0017	−0·0095	0·0061	0·6626	0·0020	−0·0023	0·0062	0·3552	−0·0047	−0·0100	0·0005	0·0772
	3	−0·0005	−0·0085	0·0076	0·9113	0·0023	−0·0020	0·0066	0·2860	−0·0057	−0·0109	−0·0004	**0·0349**
LDL-cholesterol (mmol/l)	1	−0·0011	−0·0075	0·0053	0·7385	0·0016	−0·0022	0·0054	0·3943	−0·0062	−0·0113	−0·0010	**0·0198**
	3	0·0003	−0·0065	0·0070	0·9364	0·0020	−0·0019	0·0059	0·3038	−0·0070	−0·0122	−0·0018	**0·0089**
HDL-cholesterol (mmol/l)	1	−0·0004	−0·0021	0·0014	0·6827	0·0003	−0·0009	0·0015	0·6311	−0·0012	−0·0024	−0·0001	**0·0367**
	3	−0·0016	−0·0034	0·0001	0·0628	−0·0002	−0·0014	0·0009	0·6842	−0·0011	−0·0023	0·0002	0·0875
Fasting TAG (mmol/l)	1	−0·0005	−0·0039	0·0029	0·7792	−0·0004	−0·0028	0·0020	0·7431	0·0042	0·0015	0·0069	**0·0030**
	3	0·0025	−0·0007	0·0056	0·1268	0·0009	−0·0014	0·0031	0·4396	0·0037	0·0011	0·0063	**0·0062**
Diastolic BP (mmHg)	1	−0·1131	−0·1834	−0·0428	**0·0021**	−0·0333	−0·0924	0·0258	0·2643	−0·0306	−0·0907	0·0295	0·3125
	3	−0·0589	−0·1274	0·0096	0·0908	−0·0075	−0·0638	0·0488	0·7912	−0·0336	−0·0876	0·0205	0·2190
Systolic BP (mmHg)	1	−0·0012	−0·0020	−0·0005	**0·0026**	−0·0004	−0·0011	0·0002	0·1654	−0·0004	−0·0012	0·0004	0·3624
	3	−0·0009	−0·0016	−0·0001	**0·0293**	−0·0003	−0·0009	0·0003	0·3607	−0·0004	−0·0012	0·0003	0·2377
Apo B (g/l)	1	0·0002	−0·0020	0·0023	0·8738	0·0007	−0·0007	0·0021	0·3070	−0·0004	−0·0020	0·0012	0·6387
	3	0·0012	−0·0010	0·0034	0·2817	0·0011	−0·0003	0·0025	0·1213	−0·0008	−0·0024	0·0008	0·3346
Fasting blood glucose (mmol/l)	1	−0·0001	−0·0007	0·0006	0·8006	−0·0004	−0·0008	0·0000	**0·0356**	0·0009	0·0003	0·0014	**0·0023**
	3	0·0005	−0·0001	0·0012	0·1228	−0·0002	−0·0006	0·0002	0·3820	0·0008	0·0003	0·0013	**0·0014**
	5	0·0005	−0·0002	0·0012	0·1392	−0·0002	−0·0006	0·0003	0·4532	0·0009	0·0004	0·0014	**0·0003**
HbA1c (%)	1	0·0001	−0·0004	0·0007	0·6081	0·0000	−0·0004	0·0004	0·9540	0·0004	0·0000	0·0008	**0·0333**
	3	0·0005	−0·0001	0·0011	0·1111	0·0001	−0·0003	0·0005	0·5080	0·0003	−0·0001	0·0008	0·1155
	5	0·0005	−0·0001	0·0011	0·1381	0·0001	−0·0003	0·0005	0·5054	0·0004	−0·0001	0·0008	0·1148
Waist circumference (cm)	1	−0·0017	−0·0025	−0·0008	**0·0002**	−0·0006	−0·0013	0·0000	0·0568	−0·0005	−0·0014	0·0005	0·3331
	2	−0·0015	−0·0023	−0·0007	**0·0006**	−0·0004	−0·0011	0·0002	0·2077	−0·0006	−0·0016	0·0003	0·1827
	4	−0·0015	−0·0024	−0·0006	**0·0009**	−0·0004	−0·0010	0·0002	0·2076	−0·0006	−0·0016	0·0003	0·1622
BMI (kg/m^2^)	1	−0·0030	−0·0042	−0·0019	**<0·0001**	−0·0012	−0·0021	−0·0003	**0·0096**	−0·0002	−0·0014	0·0010	0·7330
	2	−0·0029	−0·0041	−0·0018	**<0·0001**	−0·0010	−0·0019	−0·0001	**0·0313**	−0·0003	−0·0016	0·0009	0·5921
	4	−0·0029	−0·0041	−0·0017	**<0·0001**	−0·0011	−0·0020	−0·0002	**0·0185**	−0·0005	−0·0016	0·0007	0·4221

Apo, apo; BP, blood pressure; PDI, plant-based diet index; TAG, fasting TAG.

* *N* 8 769 986 (unweighted *n* 2060). Sampling and replicate weights used to generalise the results to the Australian population at the time of the survey.

† Bolded *P* values denotes those that are statistically significant *P* < 0.05.

Model 1 – Survey linear regression adjusting for age and sex

Model 2 – Survey linear regression adjusting for age, sex, education, physical activity and smoking status.

Model 3 – Survey linear regression adjusting for age, sex, education, physical activity, smoking status and BMI.

Model 4 – Survey linear regression adjusting for age, sex, education, physical activity, smoking status, intakes of energy and alcohol.

Model 5 – Survey linear regression adjusting for age, sex, education, physical activity, smoking status, BMI, intakes of energy and alcohol.

## Discussion

According to the findings of this study, a higher uPDI score was associated with 3·7 % higher odds of moderate to severe CKD, as well as increased fasting blood TAG and glucose concentrations. A high uPDI score indicated a diet richer in refined grains, added sugars and salty plant food ingredients, mainly from discretionary foods, but lower in healthy plant and animal food products. These results emphasise the importance of maintaining overall diet quality, even in a context where higher amounts of plant-based foods are consumed. This is particularly important considering the growing trend towards plant-based diets and consumption of plant-based meat alternatives. Since moderate to severe stages of CKD may require additional dietary modifications, such as changes to potassium, Na, protein and phosphate intake, our results underscore the importance of obtaining dietetic advice on how to maintain adequate diet quality.

Our findings are consistent with prior work reporting poorer health outcomes associated with unhealthy plant-based diets^(13,28,29)^. Results of the USA Atherosclerosis Risk and Communities Study that investigated 14 868 adults for 24 years found that a greater uPDI score was associated with an increased risk of CKD^(13)^. In another large study involving the South Korean adult population, a higher uPDI score was associated with increased odds of metabolic syndrome and conditions such as hypertriacylglycerolaemia, high fasting glucose and abdominal obesity^(28)^. The positive associations between the uPDI, CKD prevalence, blood TAG and glucose concentrations found in our study might be explained by mechanisms involving food components within this dietary pattern that elicit physiological responses with chronic consumption^(30)^. Consistently higher intakes of poor quality, refined or processed plant foods likely displace nutritionally superior foods in the diet, potentially leading to the depletion of essential nutrients being consumed^(31)^. This was validated in our study population, where individuals who scored higher using the uPDI had significantly lower intakes of several essential nutrients, including dietary fibre, Mg and unsaturated fats. Substantial evidence has been documented linking these dietary components with related conditions to CKD, including diabetes, dyslipidaemia and metabolic syndrome. For example, lower intakes of dietary fibre coupled with unsuitable fat composition provided by greater consumption of less healthy plant foods have been suggested to contribute to dyslipidaemia^(29)^. Excess intakes of plant food sources high in added sugar have also been known to exacerbate insulin resistance, increasing the risk of hypertriglyceridaemia and abdominal obesity^(31)^. On the other hand, low intakes of essential nutrients from health-promoting plant foods, such as Mg, have been identified as risk factors for chronic conditions^(32,33)^. Mg, which is commonly found in green leafy vegetables, legumes, nuts and seeds, serves as a crucial cofactor necessary for the movement of glucose into the cell and carbohydrate metabolism^(33)^. Research has demonstrated that adequate Mg intake reduces the risk of T2DM and metabolic syndrome by alleviating insulin resistance^(32)^.

The present study found no significant associations between the overall PDI, hPDI score and CKD prevalence or severity. While this was unexpected, there are several plausible reasons for this. Differences in dietary intakes that the overall PDI and hPDI can capture may be less pronounced in the Australian population. For example, prior investigations using the same study population found that < 4 % of adults met the minimum Australian Dietary Guidelines recommended number of serves of vegetables^(34)^. Therefore, even with the relative scoring approach employed, it may be possible that those with higher scores in this particular sample population still might have had lower healthy plant intakes, making real intakes not have been high enough to detect inverse associations between PDI, hPDI and CKD. Another possibility suggested by this finding is that habitual diets of people already diagnosed with moderate-severe CKD may be poorer, perhaps as a consequence of following restricted nutrient-based advice for people with CKD. Finally, most studies that reported a positive association between hPDI and CKD have been longitudinal^(6,13)^. Given that CKD has a long lead-time in terms of development, we considered it worthwhile to investigate dietary patterns in those with conditions at high risk of CKD or with early signs. Consistent with prior findings, our results demonstrated significant inverse associations between BMI, waist circumference and blood pressure measures with the overall PDI and the hPDI^(35)^.

This study builds upon prior research by comprehensively assessing plant-based diets by considering total plant and animal intakes from single, multi-ingredient and mixed dishes. Further strengthening the study design was the use of data from a nationally representative sample, which maximises the generalisability of our findings, as were the rigorous adjustments for potential confounders in the interpretation of data. A limitation of the 24-h recall method is that it only offers a snapshot of dietary intakes and may not fully reflect a person’s usual eating habits. To obtain a more accurate representation of usual intake, it is recommended to complete four to eight repeat 24-h recalls^(36)^, whereas the NPPAS survey only uses 2 days^(37)^. However, compared with previous studies based on one day of dietary recall, the present analysis used two 24-h recalls offering an advantage to improve accuracy and better account for such variation in intakes. The plant-based diet quality indices used in this study are based on a sample-scoring method. This means that the scores are calculated based on the diets of participants included in each study and may not be comparable across different studies. However, the indices allow us to examine the differences in proportions and increments between the highest and lowest consumers in the sample. To make comparisons easier, details of each quintile in each index are provided, including information on servings of plant and animal foods, as well as nutrient information. Finally, the cross-sectional design of this study prohibits the interpretation of causation between plant-based diets, CKD risk and related conditions and is considered a limitation.

According to this study, diets characterised by higher intakes of unhealthy plant foods, such as refined grains, salty plant products and added sugars, especially those obtained from discretionary choices, are associated with increased prevalence of CKD and elevated fasting blood TAG and glucose concentrations. In the Australian population, attention to diet quality remains paramount, even in those with higher intakes of plant foods and who wish to reduce risk of CKD. Given the additional dietary modifications that may be indicated in moderate to severe stages of CKD, it is crucial to prioritise overall diet quality thorough adequate dietary assessment and counselling. This ensures that the translation of nutritional guidelines for CKD avoids the erroneous exclusion of healthy plant foods and the substitution of less healthy options. Expanding beyond considerations of human health, prior research undertaken by our research team has also demonstrated that the environmental impact of the usual renal diet is high, and modifications to reduce this are possible and achievable^(38)^. Healthy plant-based diets with a low content of ultra-processed foods are not only healthy for individuals with CKD but they are also sustainable^(39)^.

## Supporting information

Stanford et al. supplementary material Stanford et al. supplementary material

## Data Availability

Accessibility and dissemination of the ABS Basic CURF data, obtained in the NNPAS, is governed by the Census and Statistics Act 1905(41), such that data must remain de-identified. Permission to use and analyse the data was granted through approved registration at the ABS registration centre and the University of Wollongong.

## Supplementary material

For supplementary material accompanying this paper visit https://doi.org/10.1017/S1368980024001095

## References

[1] Hill NR , Fatoba ST , Oke JL et al. (2016) Global prevalence of chronic kidney disease – a systematic review and meta-analysis. PLOS ONE 11, e0158765. 27383068 10.1371/journal.pone.0158765 PMC4934905

[2] Chen T & Harris DC (2015) Challenges of chronic kidney disease prevention. Med J Aust 203, 209–210. 26852046 10.5694/mja15.00241

[3] Australian Institute of Health and Welfare (2017) Chronic kidney disease compendium; Available at https://www.aihw.gov.au/reports/chronic-kidney-disease/chronic-kidney-disease-compendium/contents/how-many-australians-have-chronic-kidney-disease (accessed April 2018).

[4] Lecamwasam A , Ekinci EI , Richard JM et al. (2017) The threat among us: significance and scale of diabetic chronic kidney disease in Australia. Internal Med J 47, 1339–1341. 29224213 10.1111/imj.13640

[5] Australian Institute of Health and Welfare (2019) Chronic Kidney Disease. https://www.aihw.gov.au/reports-data/health-conditions-disability-deaths/chronic-kidney-disease/about (accessed March 2022).

[6] Kelly JT , Palmer SC , Wai SN et al. (2017) Healthy dietary patterns and risk of mortality and ESRD in CKD: a meta-analysis of cohort studies. Clin J Am Soc Nephrol: CJASN 12, 272–279. 27932391 10.2215/CJN.06190616 PMC5293335

[7] Benatar JR & Stewart RAH (2018) Cardiometabolic risk factors in vegans; a meta-analysis of observational studies. PLoS One 13, e0209086. 30571724 10.1371/journal.pone.0209086 PMC6301673

[8] Kim H , Caulfield LE , Garcia-Larsen V et al. (2019) Plant-based diets are associated with a lower risk of incident cardiovascular disease, cardiovascular disease mortality, and all-cause mortality in a general population of middle-aged adults. J Am Heart Assoc 8, e012865. 31387433 10.1161/JAHA.119.012865 PMC6759882

[9] Newby PK , Tucker KL & Wolk A (2005) Risk of overweight and obesity among semivegetarian, lactovegetarian, and vegan women. Am J Clin Nutr 81, 1267–1274. 15941875 10.1093/ajcn/81.6.1267

[10] Spencer EA , Appleby PN , Davey GK et al. (2003) Diet and body mass index in 38000 EPIC-Oxford meat-eaters, fish-eaters, vegetarians and vegans. Int J Obesity Relat Metab Disorders: Journal of the International Association for the Study of Obesity 27, 728–734. 10.1038/sj.ijo.0802300 12833118

[11] Kelly JT , Rossi M , Johnson DW et al. (2017) Beyond sodium, phosphate and potassium: potential dietary interventions in kidney disease. Semin Dialysis 30, 197–202. 10.1111/sdi.12580 28239979

[12] Carrero JJ , González-Ortiz A , Avesani CM et al. (2020) Plant-based diets to manage the risks and complications of chronic kidney disease. Nat Rev Nephrol 16, 525–542. 32528189 10.1038/s41581-020-0297-2

[13] Kim H , Caulfield LE , Garcia-Larsen V et al. (2019) Plant-based diets and incident CKD and kidney function. Clin J Am Soc Nephrol 14, 682. 31023928 10.2215/CJN.12391018 PMC6500948

[14] Hu EA , Steffen LM , Grams ME et al. (2019) Dietary patterns and risk of incident chronic kidney disease: the atherosclerosis risk in communities study. Am J Clin Nutr 110, 713–721. 31386145 10.1093/ajcn/nqz146 PMC6736122

[15] Liu HW , Tsai WH , Liu JS et al. (2019) Association of vegetarian diet with chronic kidney disease. Nutrients 11, 279. 30691237 10.3390/nu11020279 PMC6412429

[16] Gutiérrez OM , Muntner P , Rizk DV et al. (2014) Dietary patterns and risk of death and progression to ESRD in individuals with CKD: a cohort study. Am J Kidney Dis 64, 204–213. 24679894 10.1053/j.ajkd.2014.02.013 PMC4111976

[17] Satija A , Bhupathiraju SN , Spiegelman D et al. (2017) Healthful and unhealthful plant-based diets and the risk of coronary heart disease in U.S. adults. J Am Coll Cardiol 70, 411–422. 28728684 10.1016/j.jacc.2017.05.047 PMC5555375

[18] Kim H , Caulfield LE & Rebholz CM (2018) Healthy plant-based diets are associated with lower risk of all-cause mortality in US adults. J Nutr 148, 624–631. 29659968 10.1093/jn/nxy019 PMC6669955

[19] Stanford J , McMahon S , Lambert K et al. (2023) Expansion of an Australian food composition database to estimate plant and animal intakes. Br J Nutr 130, 1950–1960. 37157848 10.1017/S0007114523001101 PMC10630146

[20] Australian Bureau of Statistics (2013) Australian Health Survey: Users’ Guide, 2011–13. Canberra, Australia: Australian Bureau of Statistics.

[21] Moshfegh AJ , Rhodes DG , Baer DJ et al. (2008) The US department of agriculture automated multiple-pass method reduces bias in the collection of energy intakes. Am J Clin Nutr 88, 324–332. 18689367 10.1093/ajcn/88.2.324

[22] Food Standards Australia New Zealand (2013a) AUSNUT 2011–13 Food Nutrient Database File. Canberra, Australia: Food Standards Australia New Zealand.

[23] Rossi M , Johnson DW , Xu H et al. (2015) Dietary protein-fiber ratio associates with circulating levels of indoxyl sulfate and p-cresyl sulfate in chronic kidney disease patients. Nutrition, Metabolism, Cardiovasc Dis: NMCD 25, 860–865. 10.1016/j.numecd.2015.03.015 26026209

[24] Stanford J , Charlton K , Stefoska-Needham A et al. (2021) Associations among plant-based diet quality, uremic toxins, and gut microbiota profile in adults undergoing hemodialysis therapy. J Ren Nutr 31, 177–188. 32981834 10.1053/j.jrn.2020.07.008

[25] Scialla JJ & Anderson CA (2013) Dietary acid load: a novel nutritional target in chronic kidney disease?. Adv Chronic Kidney Dis 20, 141–149. 23439373 10.1053/j.ackd.2012.11.001 PMC3604792

[26] National Health and Medical Research Council (2013) Australian Dietary Guidelines. Canberra: National Health and Medical Research Council.

[27] Australian Institute of Health and Welfare (2019) High blood pressure. https://www.aihw.gov.au/reports/risk-factors/high-blood-pressure/contents/high-blood-pressure (accessed March 2022).

[28] Kim H , Anderson CA , Hu EA et al. (2021) Plasma metabolomic signatures of healthy dietary patterns in the chronic renal insufficiency cohort (CRIC) study. J Nutr 151, 2894–2907. 34195833 10.1093/jn/nxab203 PMC8485904

[29] Song S , Lee K , Park S et al. (2021) Association between unhealthful plant-based diets and possible risk of dyslipidemia. Nutrients 13, 4334. 34959886 10.3390/nu13124334 PMC8706499

[30] Tapsell LC , Neale EP , Satija A et al. (2016) Foods, nutrients, and dietary patterns: interconnections and implications for dietary guidelines. Adv Nutr 7, 445–454. 27184272 10.3945/an.115.011718 PMC4863273

[31] DiNicolantonio JJ & Berger A. (2016) Added sugars drive nutrient and energy deficit in obesity: a new paradigm. Open Heart 3, e000469. 27547437 10.1136/openhrt-2016-000469 PMC4975866

[32] Piuri G , Zocchi M , Della Porta M et al. (2021) Magnesium in obesity, metabolic syndrome, and type 2 diabetes. Nutrients 13, 320. 33499378 10.3390/nu13020320 PMC7912442

[33] Dubey P , Thakur V & Chattopadhyay M (2020) Role of minerals and trace elements in diabetes and insulin resistance. Nutrients 12, 1864. 32585827 10.3390/nu12061864 PMC7353202

[34] Australian Bureau of Statistics (2016) Australian Health Survey: Consumption of Food Groups from the Australian Dietary Guidelines (cat no. 4364.0.55.012). Available at https://www.abs.gov.au/ausstats/abs@.nsf/Lookup/4364.0.55.012main+features12011-12#:∼:text=Adults%20(aged%2019 %20years%20and,recommended%20number%20of%20vegetable%20serves. (accessed November 2021).

[35] Chen Z , Schoufour JD , Rivadeneira F et al. (2019) Plant-based Diet and Adiposity Over Time in a Middle-aged and Elderly Population: the Rotterdam Study. Epidemiol (Cambridge, Mass) 30, 303–310. 10.1097/EDE.0000000000000961 30507650

[36] Jackson KA , Byrne NM , Magarey AM et al. (2008) Minimizing random error in dietary intakes assessed by 24-h recall, in overweight and obese adults. Eur J Clin Nutr 62, 537–543. 17375109 10.1038/sj.ejcn.1602740

[37] Bell LK , Edwards S & Grieger JA (2015) The relationship between dietary patterns and metabolic health in a representative sample of adult Australians. Nutrients 7, 6491–6505. 26251918 10.3390/nu7085295 PMC4555134

[38] Clay N , Charlton K , Stefoska-Needham A et al. (2023) What is the climate footprint of therapeutic diets for people with chronic kidney disease? Results from an Australian analysis. J Hum Nutr Diet 36, 2246–2255. 37427492 10.1111/jhn.13204

[39] Avesani CM , Cardozo L , Yee-Moon Wang A et al. (2023) Planetary health, nutrition, and chronic kidney disease: connecting the dots for a sustainable future. J Ren Nutr 33, S40–S8. 36182058 10.1053/j.jrn.2022.09.003

